# Supplementary material for: USP15 Represses Hepatocellular Carcinoma Progression by Regulation of Pathways of Cell Proliferation and Cell Migration: A System Biology Analysis
Source: Cancers (Basel). 2023 Feb 21;15(5):1371. doi: 10.3390/cancers15051371 (PMC10000201; doi:10.3390/cancers15051371)
Supplement: Supplementary file 1 [file cancers-15-01371-s001.zip › cancers-2156405-Supplementary Figures.pdf]

Supplemental Figures

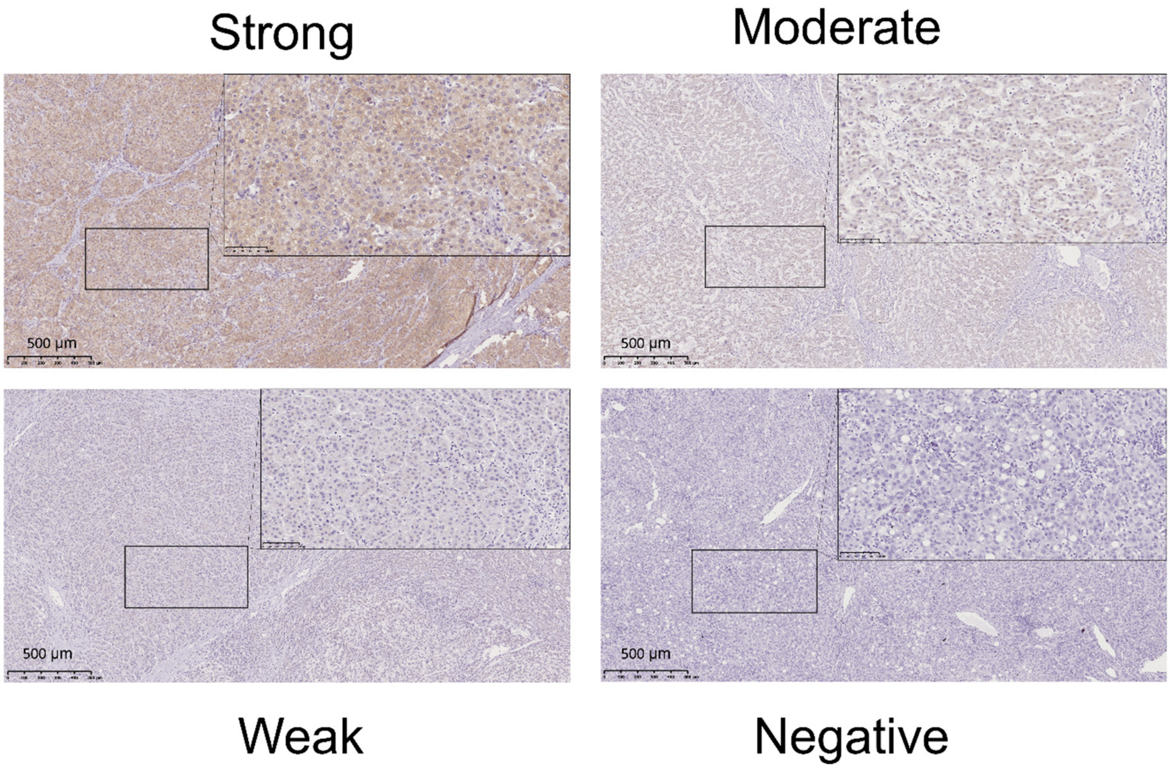

Figure S1. Reference standards of the classification of tissue. The expression level of tissue was scored based on its IHC staining for USP15. By visual inspection, two trained pathologists classified the tissue samples of 102 patients into one of the four groups: strong, moderate, weak, and negative. The four images above define the reference standards to assists the assignment of a tissue sample to a group.

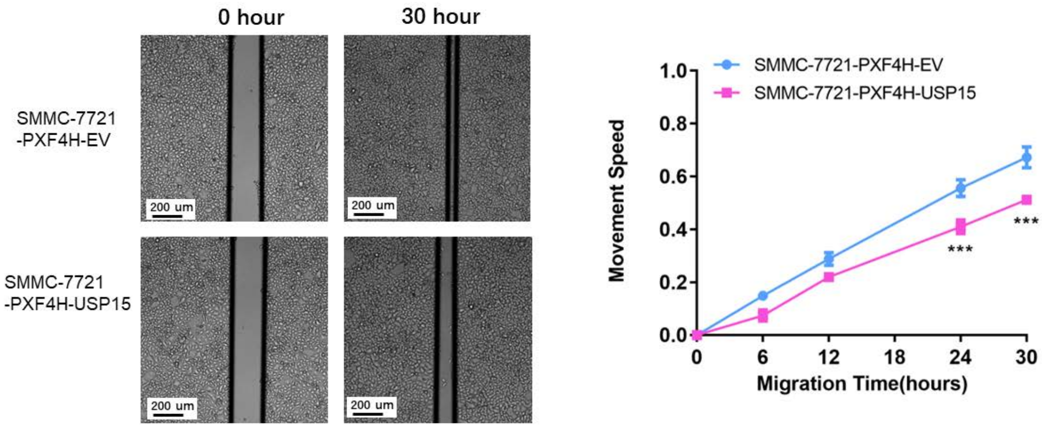

Figure S2. Wound healing assay for cell line SMMC-7721. Cells were transfected to overexpress USP 15. Overexpressing cells, SMMC-7721-USP15, have a decreased proliferation rate

compared to the control, SMMC-7721-PXF4H-EV, cells transfected with an empty vector (*** $p < 0.001$, two tailed t-test)

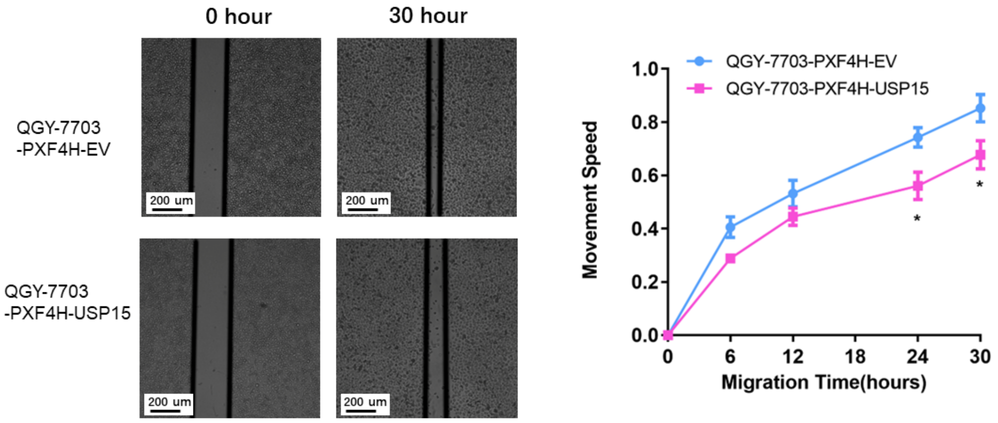

Figure S3. Wound healing assay for cell line QGY-7703. Cells were transfected to overexpress USP 15. Overexpressing cells, QGY-7703 -USP15, have a decreased proliferation rate compared to the control, QGY-7703 -PXF4H-EV, cells transfected with an empty vector (* $p < 0.05$, two tailed t-test)

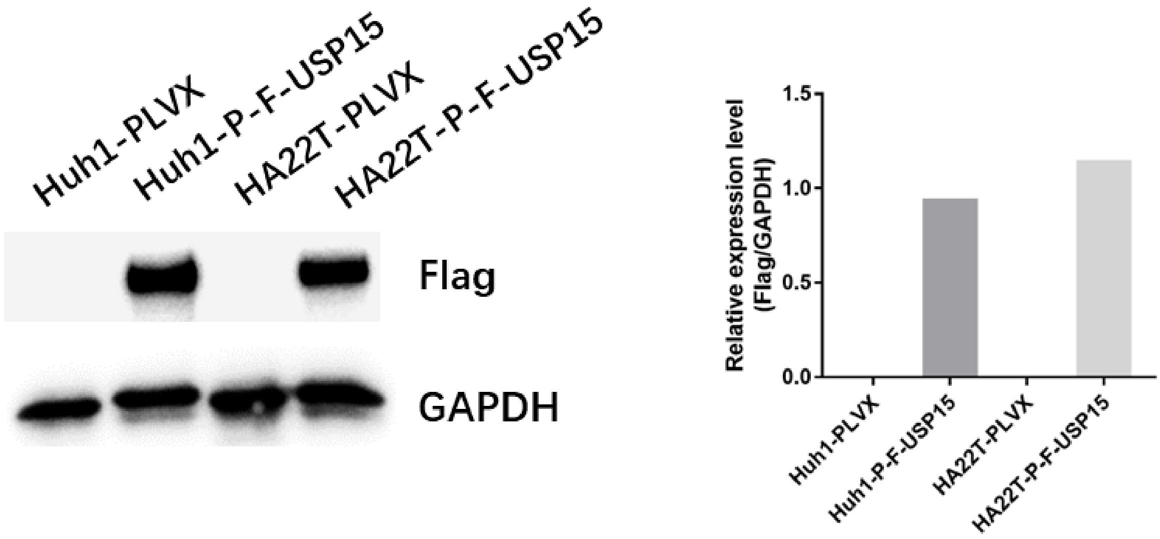

Figure S4. Overexpression efficiency of the plasmids for PF-USP15 Huh1 and HA22T. The right picture is the band densitometry intensity ration of the western blot of the left picture.

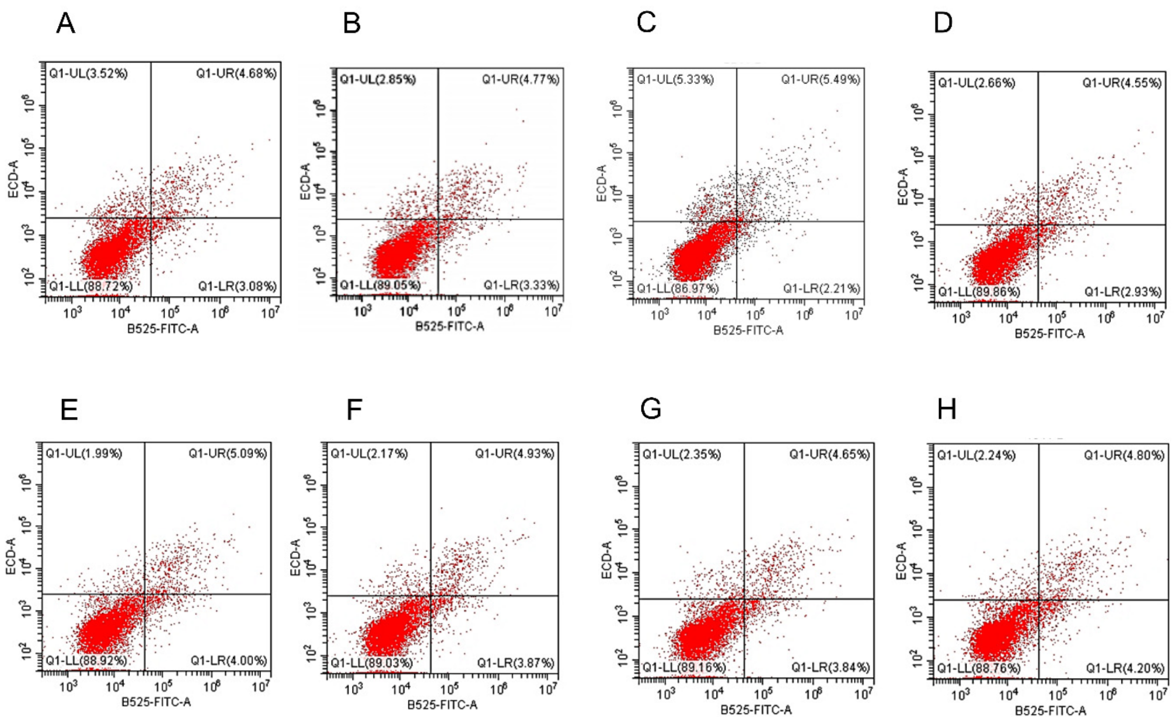

Figure S5. (**A**) Flow cytometery for HA22T SG-control (empty vector transfected into the cells). (**B**) Flow cytometery for HA22T with USP15 knockout. (**C**) Flow cytometery for Huh1 SG-control (empty vector transfected into the cells). (**D**) Flow cytometery for Huh1 with USP15 knockout. (**E**) Flow cytometery for HA22T OE-control (empty vector transfected into the cells). (**F**) Flow cytometery for HA22T with USP15 overexpression. (**G**) Flow cytometery for Huh1 OE-control (empty vector transfected into the cells). (**H**) Flow cytometery for Huh1 with USP15 overexpression.

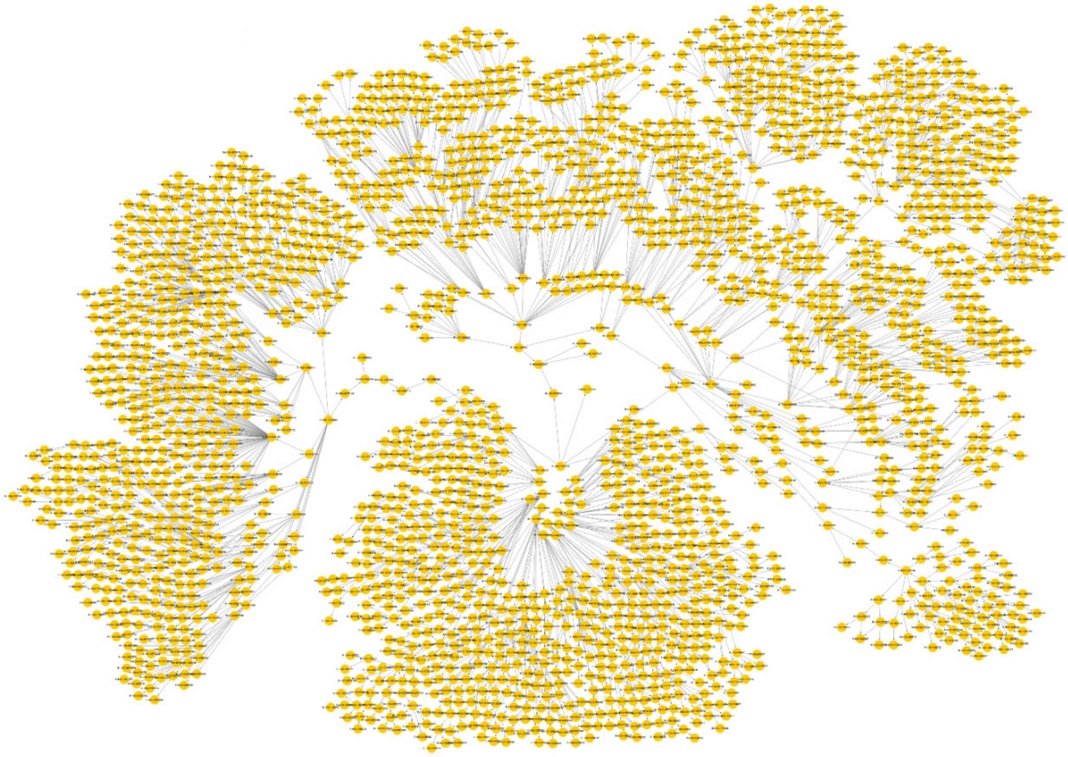

Figure S6. Global view of the hierarchy relationships of whole human pathways. Used as the blank control for Figure 5C.

Figure 2A

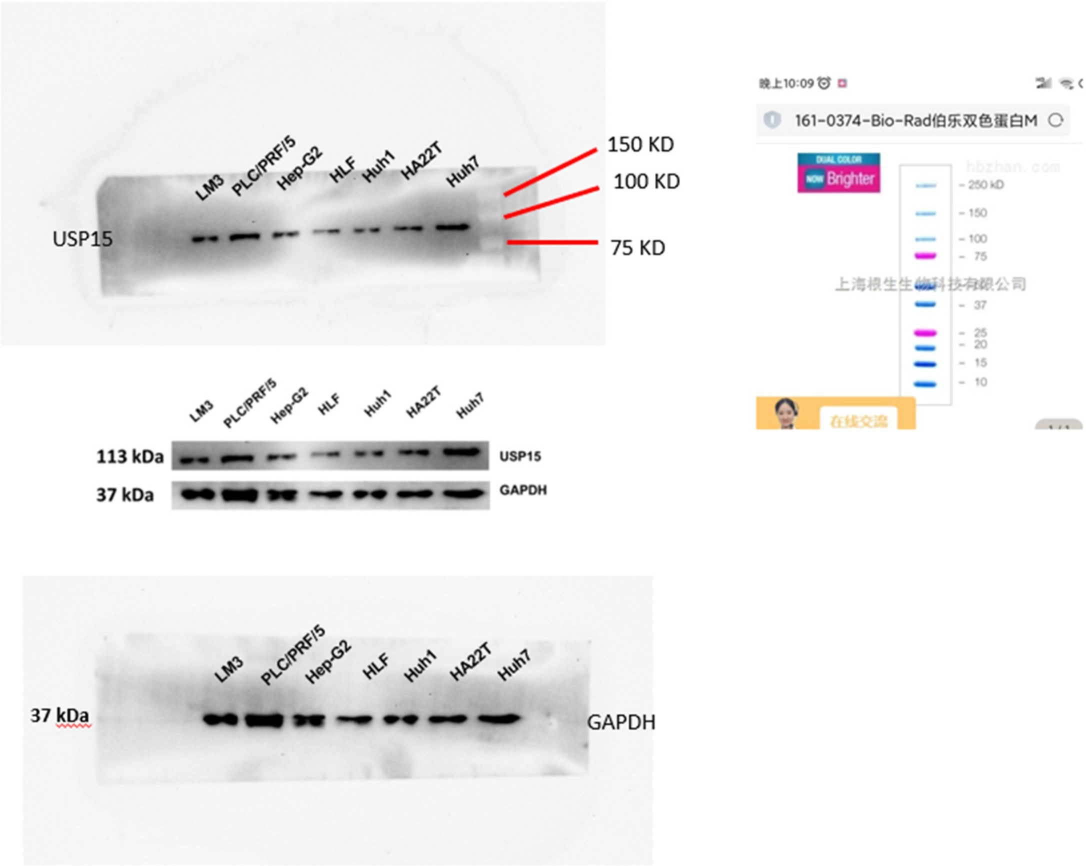

Figure 2I

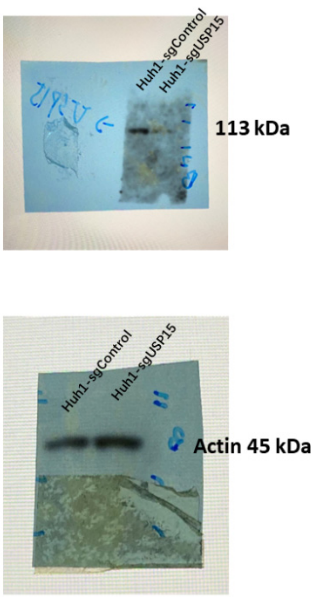

Figure 2M

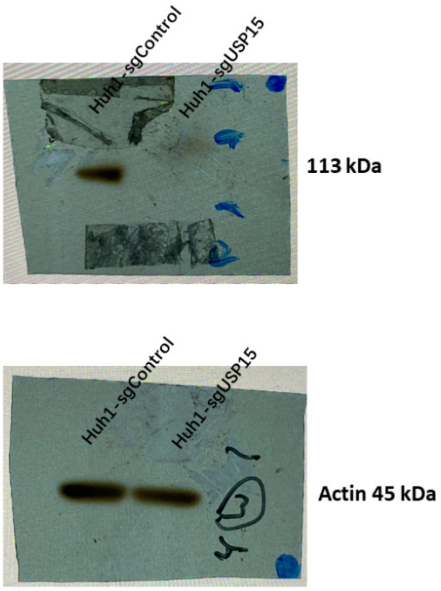

Figure 4A

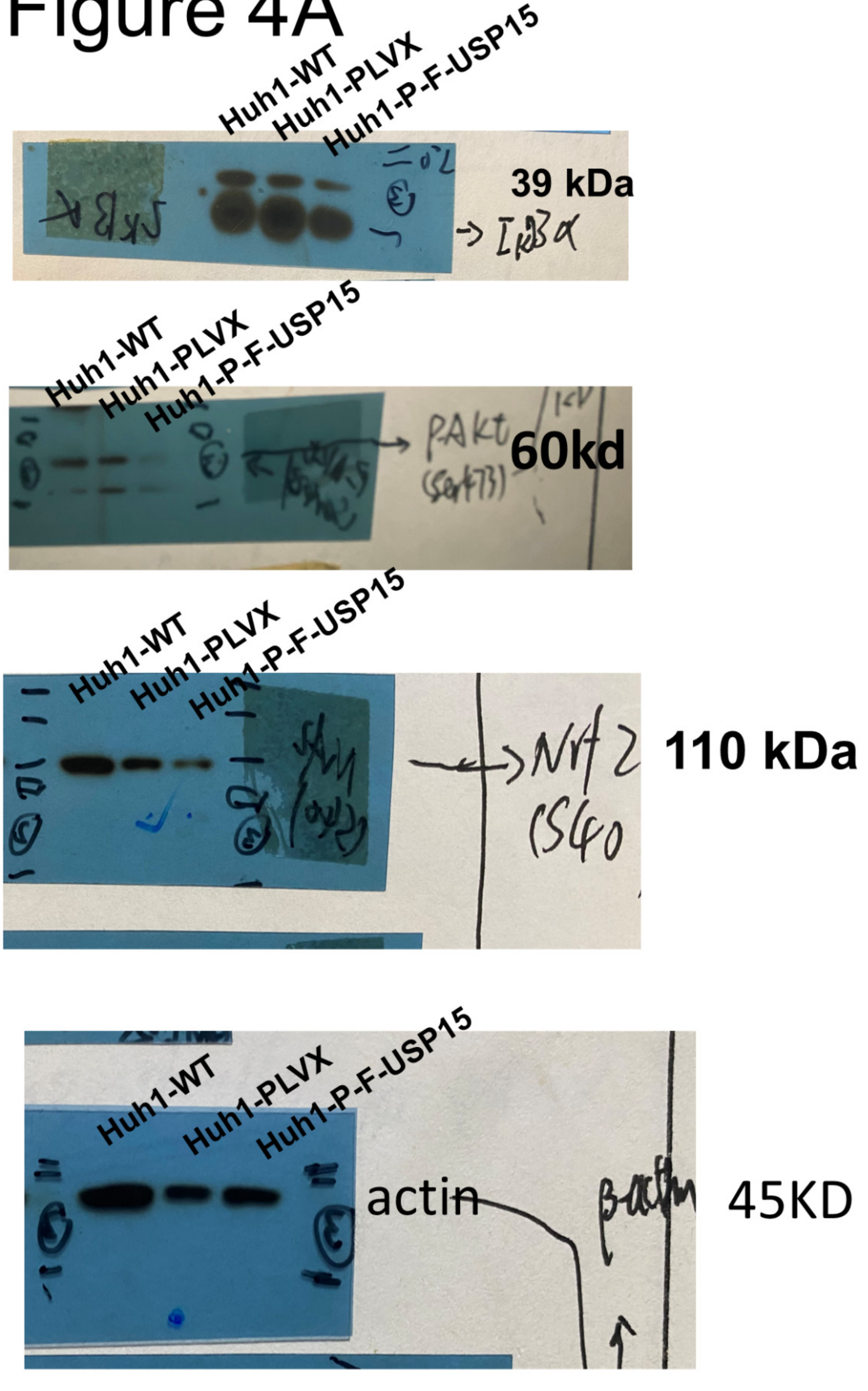
Huh1-WT
Huh1-PLVX
Huh1-P-F-USP15
39 kDa
Huh1-WT
Huh1-PLVX
Huh1-P-F-USP15
60kd
Huh1-WT
Huh1-PLVX
Huh1-P-F-USP15
110 kDa
Huh1-WT
Huh1-PLVX
Huh1-P-F-USP15
actin
45KD

Figure 4B

pERK 42KD

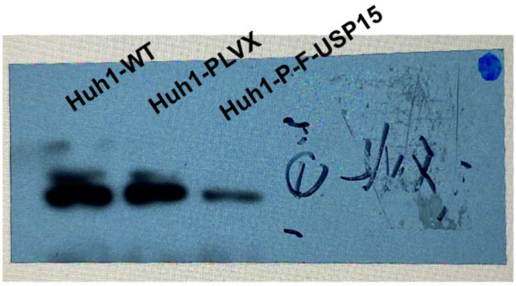

β-catenin 92KD

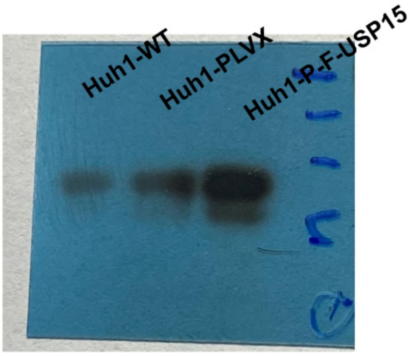

actin 45KD

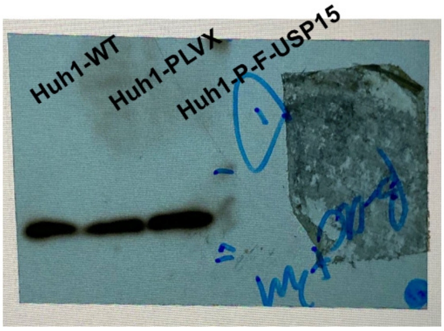

p-Smad 60kDa

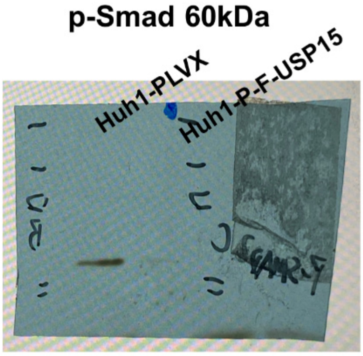

actin 45KD

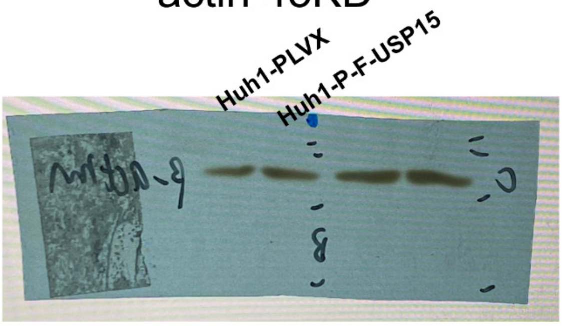

Figure 4C

pAKT 60KD

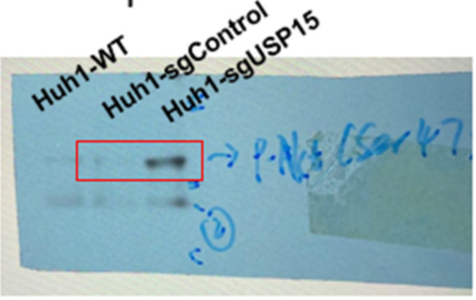

IKBA 39KD

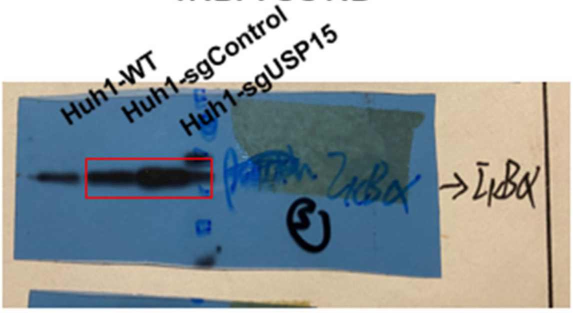

pNrf2 110KD

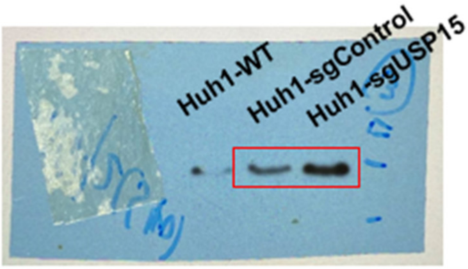

pRb1 110KD

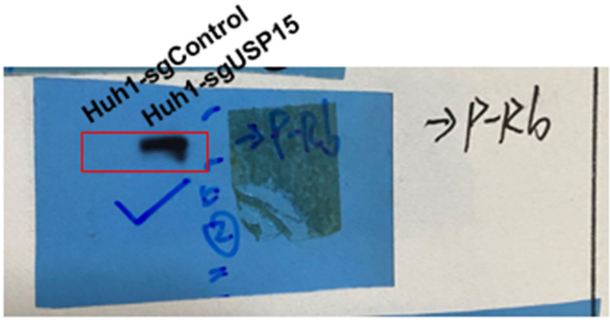

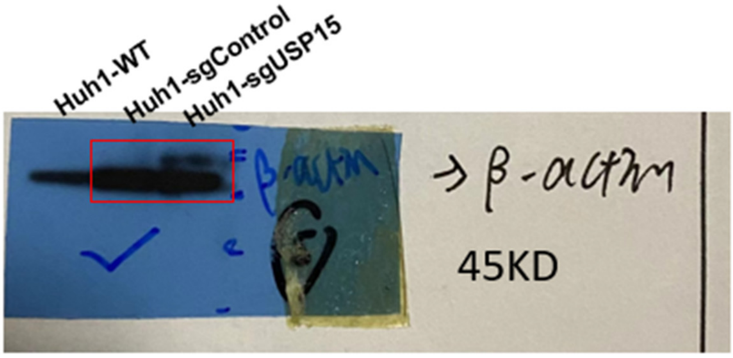

Figure 4D

c-myc 57-65KD

Huh1-sgControl
Huh1-sgUSP15

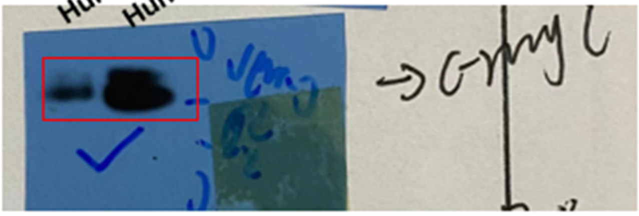

β-catenin 92KD

Huh1-sgControl
Huh1-sgUSP15

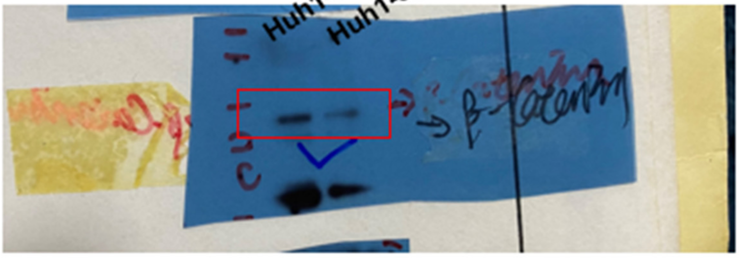

Huh1-sgControl
Huh1-sgUSP15

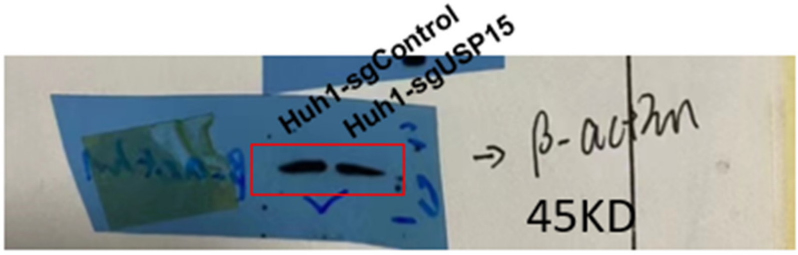

45KD

Figure 4E

N-cadherin 110KD

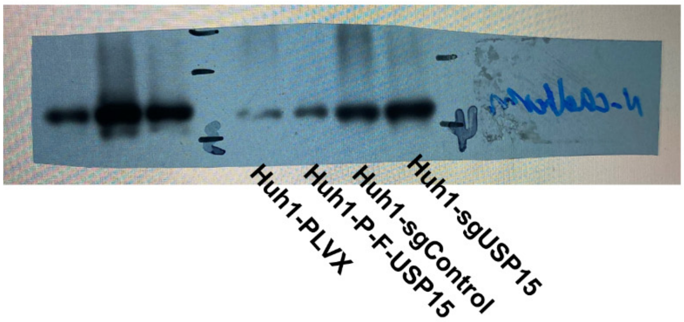

actin 45KD

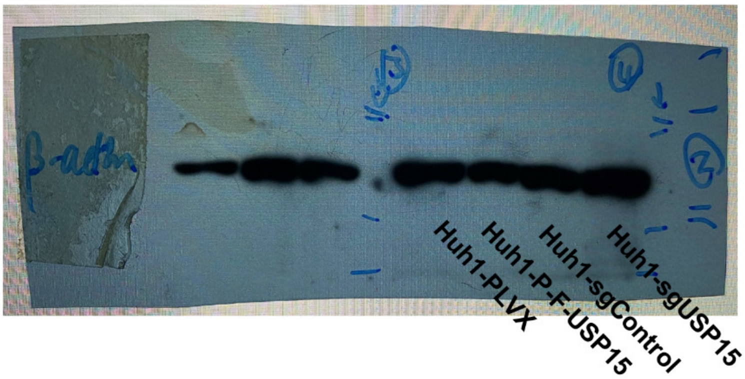

Figure 4F

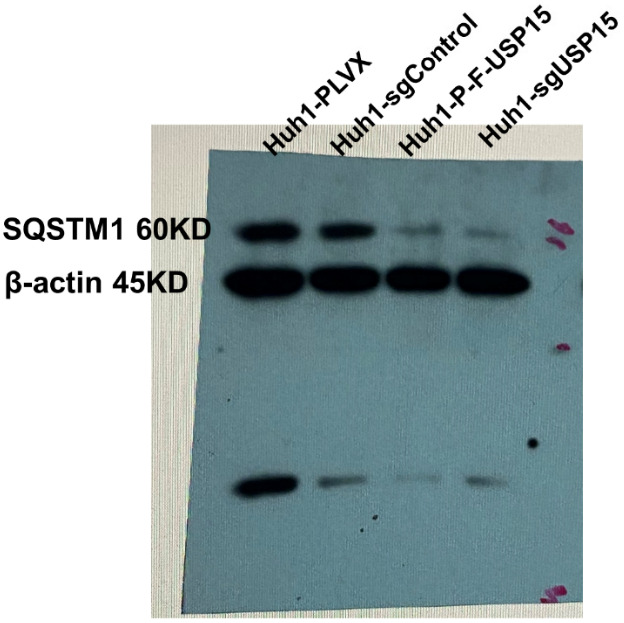

Figure 4J

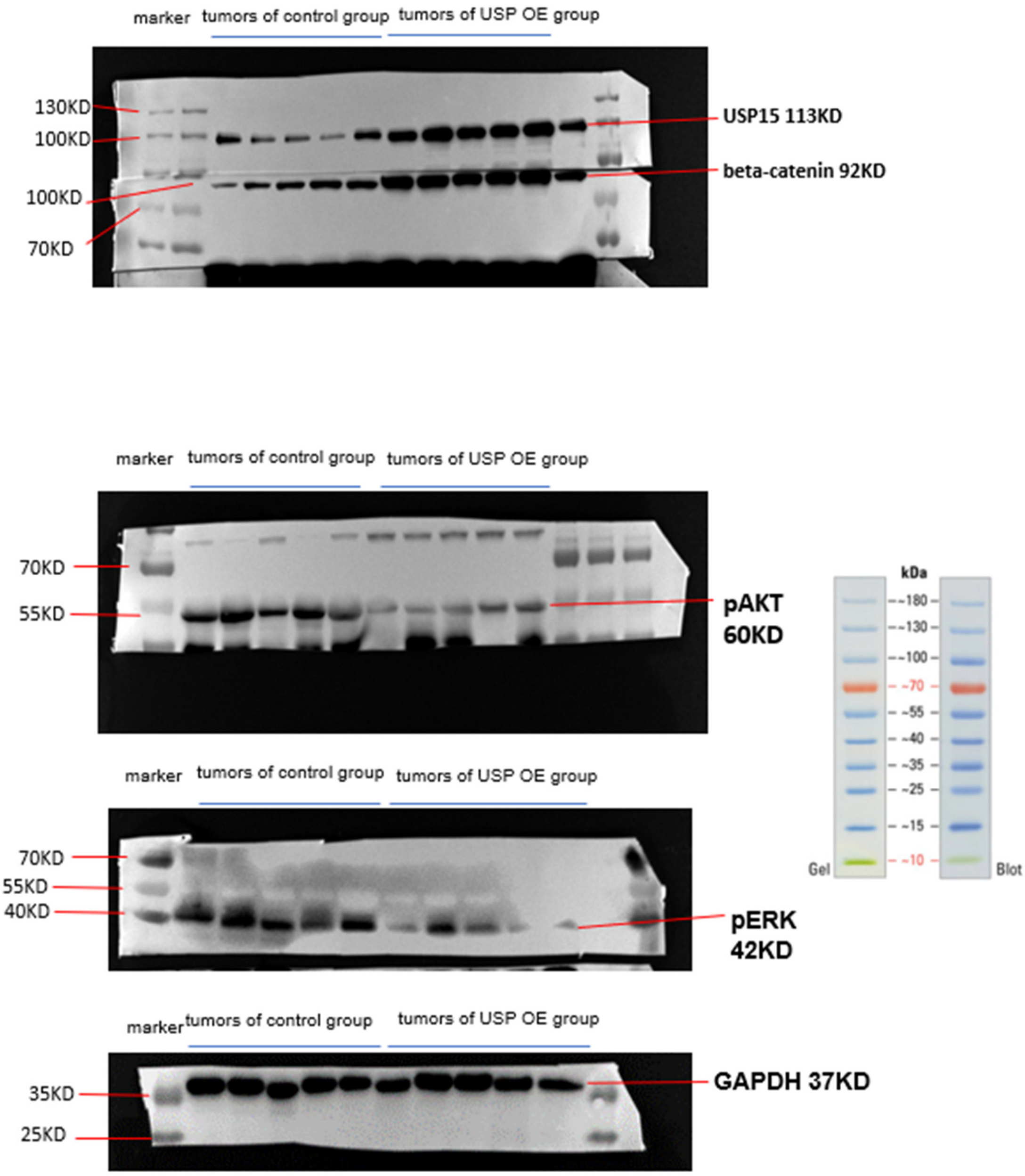

marker
tumors of control group
tumors of USP OE group
130KD
100KD
100KD
70KD
USP15 113KD
beta-catenin 92KD
marker
tumors of control group
tumors of USP OE group
70KD
55KD
pAKT 60KD
marker
tumors of control group
tumors of USP OE group
70KD
55KD
40KD
pERK 42KD
marker
tumors of control group
tumors of USP OE group
35KD
25KD
GAPDH 37KD
kDa
~180
~130
~100
~70
~55
~40
~35
~25
~15
~10
Gel
Blot

Figure S4

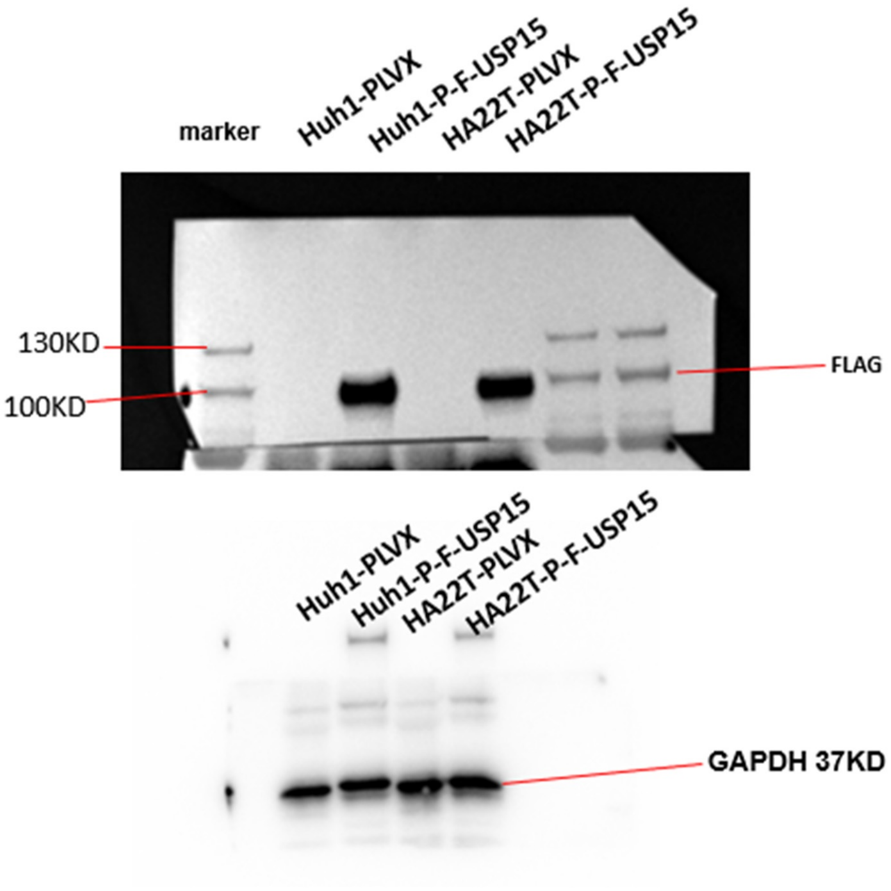

Figure S7. Original Western Blot images.
